# Hygiene guideline for the planning, installation, and operation of ventilation and air-conditioning systems in health-care settings – Guideline of the German Society for Hospital Hygiene (DGKH)

**DOI:** 10.3205/dgkh000263

**Published:** 2016-02-16

**Authors:** Rüdiger Külpmann, Bärbel Christiansen, Axel Kramer, Peter Lüderitz, Frank-Albert Pitten, Frank Wille, Klaus-Dieter Zastrow, Friederike Lemm, Regina Sommer, Milo Halabi

**Affiliations:** 1 German Society for Hospital Hygiene (DGKH), Berlin, Germany; 2 Working Group Hygiene in Hospital and Practice, Association of the Scientific Medical Societies (AWMF), Germany; 3 Austrian Society of Hygiene, Microbiology and Preventive Medicine (ÖGHMP), Vienna, Austria

## Abstract

Since the publication of the first “Hospital Hygiene Guideline for the implementation and operation of air conditioning systems (HVAC systems) in hospitals” (http://www.krankenhaushygiene.de/informationen/fachinformationen/leitlinien/12) in 2002, it was necessary due to the increase in knowledge, new regulations, improved air-conditioning systems and advanced test methods to revise the guideline. Based on the description of the basic features of ventilation concepts, its hygienic test and the usage-based requirements for ventilation, the DGKH section “Ventilation and air conditioning technology” attempts to provide answers for the major air quality issues in the planning, design and the hygienically safe operation of HVAC systems in rooms of health care.

## Zusammenfassung

Seit der Herausgabe der ersten „Krankenhaushygienischen Leitlinie für die Ausführung und den Betrieb von Raumlufttechnischen Anlagen (RLT-Anlagen) in Krankenhäusern“ (http://www.krankenhaushygiene.de/informationen/fachinformationen/leitlinien/12) im Jahr 2002 war es auf Grund des Wissenszuwachses, neuer Regelwerke, verbesserter Raumlufttechnischer Anlagen und weiterentwickelter Prüfverfahren erforderlich, die Leitlinie zu überarbeiten. Basierend auf der Beschreibung der Grundmerkmale von Lüftungskonzepten, ihrer hygienischen Prüfung und den nutzungsabhängigen Anforderungen an die Raumlüftung hat die DGKH-Sektion „Klima und Raumlufttechnik“ versucht, Antworten für die wesentlichen lufthygienischen Fragen bei der Planung, Auslegung und dem hygienisch sicheren Betrieb von RLT-Anlagen in Räumen des Gesundheitswesens zu geben.

## 1 Introduction

These guidelines address certain aspects of the planning, installation, and economic operation of ventilation and air-conditioning systems, namely aspects that are relevant from the perspective of hospital hygiene and infection prevention. The guidelines are based on an assessment of contemporary research and are informed by a preventive view of certain aspects of infection prophylaxis for patients, occupational health and safety, and for thermal comfort. Since hygienic testing is part of a precise operation of ventilation and air-conditioning systems, this aspect will be included as well.

First, we will list features of air conditioning that can result in different degrees of hygienic efficacy of air conditioning systems. Second, goals as well as recommended air-conditioning concepts and testing methods for health-care spaces will be listed. Additional references to current guidelines and some further suggestions are also given. 

In general, the current guidelines [1], [2], [3], [4], [5] for the planning, installation, and economic operation of ventilation and air-conditioning systems of health-care settings have to be observed.

This guide provides additional recommendations for interpreting, installing, and operating non-portable ventilation and air-conditioning systems. A separate guide provides recommendations for mobile air-cleaning and air-cooling systems.

## 2 Basic variants of air-conditioning concepts

According to the main directions of air movement, air-conditioning concepts are divided into displacement and mixed-flow ventilation systems. These systems are marked by different efficacy levels when they move air pollutants out of the room. Depending on the use of a room, various filtrations of supply air or exhaust air may be necessary and different supply air or exhaust air volume flows can help reach a defined flow from space to space. Table 1 (Tab. 1) shows three ventilation and air-conditioning concepts that differ in filtration and circulation systems and thus differ in effectiveness.

The test methods and target values serve to ascertain the hygienic effectiveness of the air-conditioning concept. 

Each air-conditioning concept has a value of its own and should be chosen accordingly.

## 3 Recommendations for user units

### 3.1 Operating room (OR) area

#### 3.1.1 Operating rooms

##### Goals

In order to protect the patients, the aerogenic influx of airborne microorganisms into the area of the OR field, on instrument tables, implants, and sterile test parts must be minimized. Personnel must be protected from airborne pollutants and the inhalation of surgical smoke. The reduction of air cleanliness because of leakage and opening doors must be minimized by directional overflow.

The room temperatures stipulated by the standards cannot do justice to all of the OR personnel because they work and must dress differently. Each institution has to reach a consensus on temperature. Hypothermia, except for when therapeutically indicated, is to be avoided pre-, intra- and post-operatively, since it is a risk factor for surgical site infections (SSI) [6], [7], [8]. There is no evidence that a specific air-conditioning concept expedites the hypothermic process. In order to avoid hypothermia, medical-technical heating systems must be used that do not reduce the air quality in the OR, e.g., warm-water systems. Active warming, e.g., pre-operative warming with a flexible warming shirt and intraoperative warming with a Bair Hugger warming unit is more effective than passive warming; a combination of both options may be good for vulnerable procedures [9].

##### Air-conditioning concepts

For ORs, the air-conditioning concepts RK (room class) Ia or RK Ib are required [1]. Research on the epidemiological benefit of RK Ia on the SSI rate has been conducted only for the implantation of alloplastic hip- and knee-joint replacements; the results are conflicting. Prospective controlled studies [10], [11] showed proof that RLTA (air conditioning systems) with LAF (laminar airflow) had a prophylactic influence on infections; however, this influence was rendered statistically irrelevant when perioperative antibiotic prophylaxis was administered [10], [11], [12]. New, albeit retrospective non-controlled studies showed no protective influence in comparison to TML (mixed flow ventilation) [13], [14], [15], [16], [17]. All studies are marked by a number of limitations [18], [19], and thus do not lead to conclusive evidence at this point in time [20].

In addition, low-grade infections have not been taken into account and therefore present a further limitation of all studies to date. Low-grade infections develop in up to one-third of the hip endoprostheses only after the regular surveillance term of one year, i.e., after >2 years [21] and they used to be considered aseptic loosening of joint implants [22].

However, the RK Ia indubitably compares favorably with any mixed ventilation system (room class Ib), i.e., physically, a significantly higher reduction rate of germ and particles loads (OR field, tables of instruments) has been proven [23], [24], [25], [26], [27], [28], [29], [30], [31], [32], [33], [34]. Therefore, from the perspective of prevention, an RK Ia OR is generally recommended for all operations that have the highest requirements for very low germ rates (e.g., implantation of large endoprotheses in the fields of orthopedics/trauma surgery). In addition, carcinogenic smoke from intraoperative combustion processes is removed faster and more effectively [35].

Both facts lead to preventive consequences that are, for instance, implemented in the aseptic production of cytostatic drugs. Their production strictly follows the principle of primary prevention, despite epidemiological evidence. For pharmacies, for example, it would be unthinkable not to prepare an aseptic medicine under clean room conditions, although, due to the comparatively short exposure time, the risk of contamination is much smaller than during implantation of a hip endoprosthesis. Along those lines and regarding the consequences for patients, it is recommended to make use of all options that prevent implant-associated infections after hip- and knee-joint replacements [36].

The characteristics of the air-conditioning concept room class Ia are influenced by the size of the ceiling field that creates a protective space, by surgical lights that should be aerodynamically optimized, and by air that should be discharged into the lower part of the room. If a coordinated choice of components is impossible, the advantages of the entire system may be lost. Tables for instruments, implants, and test parts adjacent to the OR table are protected by the adequate size of the ceiling field; a small size diminishes the protected area and reduces the diluting flow of air [30], [37], [38].

A decision on the type of air-conditioning concept should be made based on the current state of knowledge and the current and future needs of all user groups; the decision should be documented. It must be borne in mind that ORs are used for at least 20–30 years, the requirements for low germ rates will rise due to demographic developments, the number of immunocompromised patients will rise, and the number of medical interventions fraught with risk will rise as well. In addition, OR frequencies as well as OR types, institutional structures and leadership are marked by dynamic change. In light of future developments and the advantages listed above, it is recommended to equip several ORs in a new OR department with the same air-conditioning ceilings and the same surgical lights, and to subject all of the equipment to ventilation and air-conditioning tests.

In addition, the costs of the consolidated balance sheets of room class Ib and la do not differ significantly [35], [39], [40].

##### Room class Ia

A stable displacement ventilation creates a protected space in which the OR table, the OR team in sterile clothing, the sterile instruments, and other sterile tables can be placed. Pollutants emitted within this protected area are discharged directly into the back of the room by a directional flow. The entry of pollutants emitted outside of this protected area is blocked to such a degree that it not even necessary to disinfect the floor in between ophthalmological operations [29]. In this concept, a supply air flow that is virtually free of germs and particles diminishes (by diluting and discharging the air) all pollutants emitted inside the room at a high dilution speed.

*Air hygiene test:* Since the efficiency of TAV ventilation (displacement or ultra-clean ventilation) is influenced significantly by the flow permeability of the surgical lights and the arrangement of the air circulation openings, air-hygiene testing should employ the degree-of-protection method according to [1], appendix C or [5], which includes the testing of surgical lights.

##### Room class Ib

A turbulent mixed flow ventilation brings supply air into the room via terminal H13 air filters and distributes it almost homogeneously. The dilution velocity depends on the amount of air supplied into the room. This air-conditioning concept does not provide for a protected space.

*Air hygiene test:* A mixed flow ventilation does not create a protected area in the room. Therefore, an air hygiene test has to determine the time it takes for a particle load to be reduced to one hundredth of its original value (recovery time 100:1 according to [41]).

**Figure 1:**
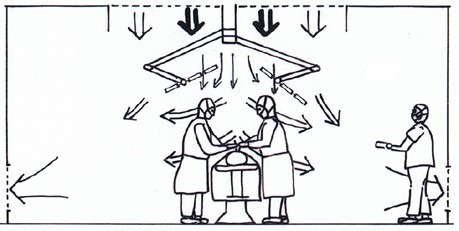
The displacement ventilation works well. The mutual enhancement of the form of the OR lamps and the location of the air circulation openings is crucial.

**Figure 2:**
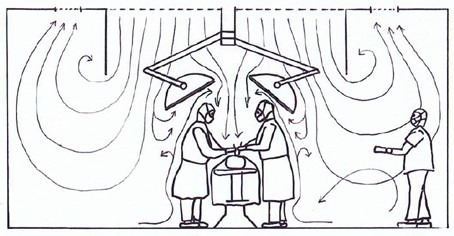
An almost ineffective displacement flow due to oversized surgical lights and air circulation openings near the TAV field on the ceiling.

**Figure 3:**
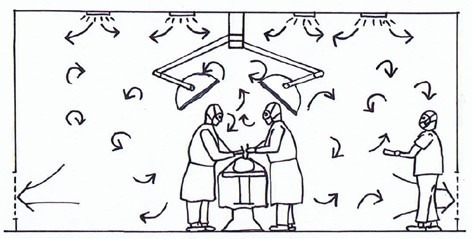
Mixed air ventilation with an air quality that is the same everywhere.

Figures 1–3 show the basic flow patterns of displacement and turbulent mixed flow ventilation. Regarding displacement ventilation, Figure 1 (Fig. 1) shows a favorable interaction of the airflow of the ceiling field with its aerodynamic surgical lights and the lower exhausts. In comparison, Figure 2 (Fig. 2) does not show an effective displacement flow, since the discharge of the supply air is blocked by large surgical lights and is fanned out by the air circulation openings on the ceiling at an early stage. The flow arrows in Figure 3 (Fig. 3) indicate that the supply air is mixed in the entire room with all emitted contaminants.

#### 3.1.2 Rooms directly adjacent to the operating room unit

##### 3.1.2.1 Anterooms

Special operations that are conducted at a high room temperature (e.g., operations on infants and burn victims) require anterooms heated to OR temperature that serve as a tempered air lock. 

##### 3.1.2.2 Preparation room for instruments 

The preparation room must have the same room-class classification as the OR for which the instruments are prepared and may only be used as a storage space for sterile goods that go into the assigned ORs. Preparatory areas protected by TAV in room class Ia must provide sufficient work space.

The temporary storage of tables that are ready for OR use requires sterile, recontamination-safe covers in the preparation room. Prepared instrument tables must not be transported through spaces of room class II (with the exception of spaces used as sterile corridors with supply air filter H13). Under these conditions, prepared instrument tables may be stored until the end of the regular business hours (max. 8 h). The activities in this room must be reduced to a minimum.

#### 3.1.3 Adjoining rooms of an operation unit

When air flows between the rooms of an operation unit, the air must only flow in the direction of the spaces that are less clean. 

The entire operation unit must be kept at a pressure differential (overpressure) to the adjoining areas connected by doors, with the cleaner areas at a higher pressure.

#### 3.1.4 Night setback or overnight shutdown

As opposed to DIN 1946-4 [1], hygiene would permit a complete shutdown of all the equipment in ORs. After the final operation and the closing of all OR doors, a shutdown time of ca. 30 min suffices. A timely restart of the equipment has to be guaranteed (e.g., when the first person enters the OR and turns on the light, the restart is launched). The recovery time must be determined as stipulated by the qualifying measurements of each OR. The recovery time must be completed before the start of the first operation; this has to be guaranteed.

For logistic or technical reasons, it might make sense to maintain air hygiene constantly in certain ORs and their preparation rooms, keeping them available for emergency purposes (in case the ORs and preparation rooms are TAV ventilated). 

### 3.2 Room for protective isolation

Air cleanliness is always defined by medical tasks, and the regulations have to be followed (in case of rooms for patients with severe burns, the regulations for room temperature and interior air humidity must also be observed).

Air hygiene classifies rooms for the protective isolation of immunosuppressed patients as room class II and adds the following requirements: In addition to the norms specified by DIN 1946-4 [1], rooms for patients of risk groups 2 and 3 must be supplied with terminal H13 filters following the KRINKO (Commission for Hospital Hygiene and Infectious Disease Prevention) suggestion [42] and must be run on higher pressure than all adjacent rooms. Window ventilation is not allowed. 

*Air hygiene test:* A test of the recovery time of 100:1 inside the room is not necessary, assuming that the room did not pollute itself. The entry of germs from the outside must be precluded. The effectiveness of the overpressure must be tested annually by testing the direction of the airflow according to DIN 1946-4 [1] 2008.

Since immunosuppressed patients cannot become infectious themselves, it is recommended to also install gates that are run on lower pressure than the patients’ rooms and the corridor. 

### 3.3 Isolation room for patients who release infectious aerosols

The goal is to protect the environment from the spread of aerosols containing highly contagious, dangerous pathogens (e.g., hemorrhagic fever, SARS, MDR tuberculosis, etc.). These rooms are classified as room class II and have a lower pressure than all adjacent rooms. Window ventilation is not allowed. Generally, the air in an upstream gate must be directed from the corridor into the patient’s room. Alternatively, for individual cases, the room opposite the patient’s room can be set on overpressure (if an extension of room usage is intended). 

If necessary, the exhaust air must be directed through an H13 filter before it is transported off into an exhaust air-duct system.

*Air hygiene test:* Periodically, the correct direction of the airflow must be verified. 

### 3.4 Emergency room

Emergency rooms are generally classified as room class II. If possible, it is recommended to keep waiting rooms at negative pressure in order to minimize the potential spread of contaminated aerosols. 

### 3.5 Intensive care (ITS), intermediate care (IMC)

These rooms are generally classified as room class II. It is recommended to keep them at negative pressure. It suffices to discharge the air of the sanitary units to prohibit aerogenic distribution between the patients’ rooms. A centralized supervision of the patients is necessary because otherwise the doors to patients’ rooms cannot be closed. Requirements for room climate properties must be met. It is to be expected that patients’ rooms are marked by a higher odor load; the air-conditioning concepts should take this into account. For intensive care units in wards that focus on treating highly immunosuppressed or infectious patients, see chapters 3.2 and 3.3.

Unclean workrooms must be supplied with a powerful exhaust air system.

### 3.6 Central sterilization

The KRINKO/BfArM (Federal Institute for Pharmaceuticals and Medical Products) guidelines for hygiene requirements for the preparation of medical products [43] are sufficient and do not place any more demands on ventilation and air conditioning. These rooms are classified as room class II.

In order to obtain a good room climate, ventilation and air conditioning are, however, necessary to discharge the heat and moisture loads from the cleaning areas and the autoclaves emptying areas.

The packaging zones must be run at higher pressure than the unclean areas. 

### 3.7 Normal wards

Normal wards do not require special ventilation and air conditioning. These rooms are classified room class II.

Unclean workrooms must be supplied with a powerful exhaust system. 

## 4 Qualification and requalification

### 4.1 Documentation of requirements

The basis of every initial qualification is a documentation of the medical service to be performed. This documentation must be written carefully, be future-oriented, and be part of the design contract.

The basis of every requalification is still the intended medical service that might have changed or not.

A hospital hygienist should be consulted for advice [44].

### 4.2 Initial qualification

#### 4.2.1 Operating rooms class Ia

The construction of the ORs for which a permit is sought (and respectively all adjacent rooms) must be totally completed. All building-technological and medical-technological equipment, the facilities and the furniture of the rooms must be present and ready for use; all building-technological installation and function tests (and respectively all rectifications) must be finished and documented.

For the initial qualification of ORs class Ia, a visual preliminary examination comes first ([1], appendix B) and the degree-of-protection method ([1], appendix C) is employed.

In order to ease the qualification and reduce time, the initial qualification may be conducted as follows: All visualizations of airflow, degree-of-protection measurements, and the definition of protected area boundaries are conducted with the surgical lights, tables, and mannequins set at the positions defined by the norms. 

If a summative degree of protection (SG) of ≥2 can be measured and if a protected area appropriate to the designated medical service can be proven, there is no need to conduct any further measurements with the surgical lights slewed out to their maximum (further measurements would be needed, however, to determine if, for instance, the protection is lowered by the air supply ceiling, the direction of air flow in the room or the surgical lights).

If a clinic uses monitors or trolleys that intrude into the protected area, the hygienist should agree with the client on an examination of the protection. At the very least, measurements should visualize the direction of the airflow and they should be documented. 

In order to reach a better understanding of how the displacement flow works, only OR personnel (head surgeons, surgeons, OR nurses) should be present during the certification (only visualization of air flow). Some ideas for the positioning of personnel, surgical lights, monitors, trolleys, and tables could then be tested *ad hoc* by trying them out. 

Microbiological processes should not be applied for an OR certification. At his or her own discretion, however, the hospital hygienist could determine the airborne germ load for didactic purposes.

#### 4.2.2 Preparation rooms for instruments for class Ia operations

The preparation space is to be tested under realistic loading conditions of loads by a video-documented visualization of air direction (min. 2 tables and one scrub nurse/surgical assistant or a heated dummy).

#### 4.2.3 Operating rooms and preparation rooms for instruments class Ib

The initial certification inspection of the OR or the preparation room for instruments is conducted with the rooms being equipped with all inventory “ready for operation” after measuring the recovery time according DIN EN ISO 14644-3 [41]. The points where the measurements are conducted should be located 30 cm above the middle of the OR table or the tables of instruments.

A testing aerosol is distributed in the room during the enrichment phase. In order to distribute the aerosol homogeneously, a powerful air circulation ventilator must be run, and should be turned off simultaneously with the aerosol generator after reaching an initial particle concentration of ca. 350,000 particles ≥0.5 µm/ft^3^ . 

The recovery time 100:1 for particles ≥0.5 µm should be measured and documented. Requirement: recovery time within 20 min.

### 4.3 Recertification

#### 4.3.1 Operating rooms class Ia and/or resp. en suite preparation rooms for instruments 

A complete visualization of airflow under load conditions has to must be conducted annually. 

A complete recertification analogous to the initial recertification is only necessary if changes are implemented that affect the air volume flows, the air flow direction in the room, the heating and/or cooling, and the installation of equipment that diverts the airflow in or under the air supply distributor. A recertification analogous to the initial certification must also be conducted when the airflow cannot be visualized properly.

Every two years, partial recertification examining the following points must be conducted:

- sealing seat and leakage of the filter for suspended matter
- grid measurement of air exit velocities of the air supply distributor, calculation of the total air supply volume flow
- measurement and documentation of the negative pressure in the confined space of the false ceiling when all doors are closed and when they are open
- the air direction must be tested at every door that is normally closed; for the test, the doors must be 1 cm ajar.

#### 4.3.2 Operating rooms and preparation rooms class Ib

At least once a year, all pressures and air directions at each door as well as at the confined space of the false ceiling must be measured and documented.

Partial recertification of the following must be conducted very two years:

- sealing seat and leakage of the filter for suspended matter
- negative pressure in the confined space of the false ceiling must be measured and documented when the doors are open and when they are closed
- the air direction must be tested at every door that is normally closed; for the test, the doors must be 1 cm ajar
- measurement of recovery time.

#### 4.3.3 Central sterilization

Every year, the air direction and the pressure must be tested at every door.

If the architecture, the facilities, and the use have not changed, technical recertification’s must be conducted every two years.

#### 4.3.4 Clearance after recertification

Any form of deficit, shortcoming, or significant deviation from the norm requires technical touch ups. The results have to be tested and documented. Only after this can clearance for use be given.

## 5 Certification of older operating rooms

### 5.1 Certification according to DIN 1946-4: 2008

Currently, many ORs built according to DIN 1946-4 are still in use.

Based on the medical services currently being performed in the OR, the user must in general first determine whether he or she wants to conduct an initial or a requalification according to chapter 5.1 or 5.2 of these guidelines. From the perspective of hygiene, the application of DIN 1946-4 [1] is recommended, since these standards do not simply test the proper functioning of filters, they also subject the entire ventilation and air-conditioning system to testing.

However, the current medical services are the basis for any certification and for the decision of whether or not to continue operating the ORs, renovate them, or downgrade them. 

If the performance of an old OR diverges in essential points from the standards formulated in DIN 1946-4 [1] concerning room class Ia, it does not make sense to employ the degree-of-protection method to qualify or requalify the OR, since one cannot assume that such an OR will pass. In this case, a recovery time measurement can be conducted as specified in 4.3.2 of these guidelines, with the goal of re-/certifying the OR as an Ib-OR. 

In consultation with the hospital hygienist, the hospital owner will decide on the spectrum of interventions in the room. 

### 5.2 Recertification according to DIN 1946-4: 1999

Routine checks according to the old DIN 1946-4 [45] standards serve primarily to demonstrate the proper functioning of the supply air filter. 

All measurements must be conducted with appropriate and calibrated instruments. The particle-measurement instruments must be able to absorb at least 28.3 l (1 ft³) per minute. In order to measure the particle concentration of the raw air in front of the filter, a dilution unit of 100:1 should be employed.

#### Particle measurements

Particle measurements have to be conducted according to DIN EN 14644-3 [41] and must render proof that the HEPA filter is tight and does not leak. 

The supply air distributors have to be dismounted such that the results of the measurements are not compromised by inducted room air. 

The particle measurements are conducted directly under the filter. According to filter class (a minimum of H13), sealing seat and non-leakage must be checked when the filter is installed. If leakage occurs, the filter must be replaced. After replacement, the new filter must also be tested.

#### Air colony count 

Air colony counts test the reduction of air colonies in the protected area. Therefore, air colony counts are not necessary underneath the supply air passages. 

#### Air flow direction

The OR must constantly have a higher pressure than the adjacent rooms and the inserted space of the false ceiling. Airflow visualization can test the pressure cascade (see 4.3).

## 6 Recommendations for training

If a hospital runs ORs of the room class Ia, its staff should be trained by a hospital hygienist or other qualified personnel. All OR personnel (head surgeons, surgeons, OR nurses, and anesthesia nurse) ought to understand the functioning and the effects of a displacement air system and therefore be invited to attend the certification tests.

For training purposes, the following should be visualized:

- the effects of positioning the OR lights differently,
- the effects of doors that are opened temporarily,
- the arrangement of tables for instruments, implants and test parts inside, at the periphery and outside of the protected area,
- the effect exerted by a ceiling-mounted supply unit that protrudes into the protected area.

## Notes

Dr. Lüderitz passed away in 2015.

## Competing interests

The authors declare that they have no competing interests.

## Figures and Tables

**Table 1:**
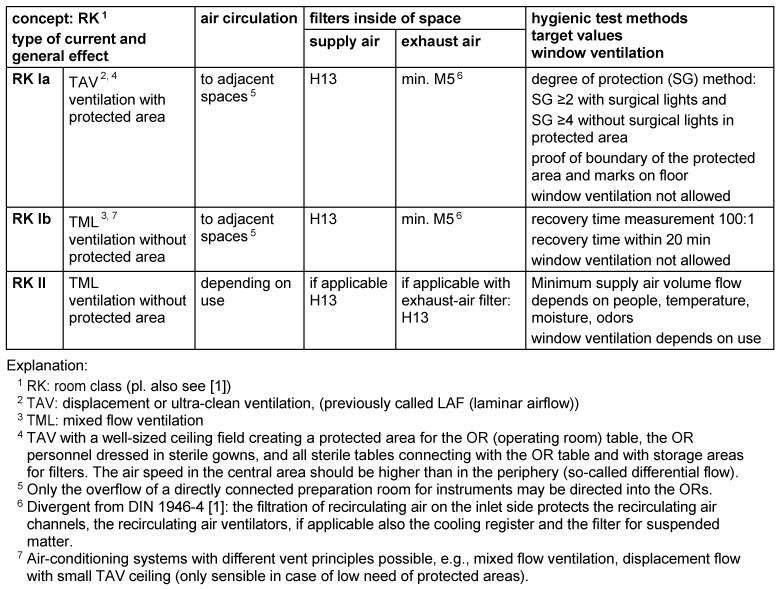
Classification, basic features, and hygienic test of air-conditioning concepts
